# Perception of Environmental Sounds in Cochlear Implant Users: A Systematic Review

**DOI:** 10.3389/fnins.2021.788899

**Published:** 2022-01-10

**Authors:** Valeriy Shafiro, Nathan Luzum, Aaron C. Moberly, Michael S. Harris

**Affiliations:** ^1^Department of Communication Disorders and Sciences, Rush University Medical Center, Chicago, IL, United States; ^2^Medical College of Wisconsin, Milwaukee, WI, United States; ^3^Department of Otolaryngology–Head and Neck Surgery, The Ohio State University Wexner Medical Center, Columbus, OH, United States; ^4^Department of Otolaryngology and Communication Sciences, Medical College of Wisconsin, Milwaukee, WI, United States

**Keywords:** cochlear implant, systematic review, perception of environmental sounds, hearing loss, auditory assessment

## Abstract

**Objectives:** Improved perception of environmental sounds (PES) is one of the primary benefits of cochlear implantation (CI). However, past research contains mixed findings on PES ability in contemporary CI users, which at times contrast with anecdotal clinical reports. The present review examined extant PES research to provide an evidence basis for clinical counseling, identify knowledge gaps, and suggest directions for future work in this area of CI outcome assessment.

**Methods:** Six electronic databases were searched using medical subject headings (MeSH) and keywords broadly identified to reference CI and environmental sounds. Records published between 2000 and 2021 were screened by two independent reviewers in accordance with the Preferred Reporting Items for Systematic Reviews and Meta-Analysis (PRISMA) statement to identify studies that met the inclusion criteria. Data were subsequently extracted and evaluated according to synthesis without-meta-analysis (SWiM) guidelines.

**Results:** Nineteen studies met the inclusion criteria. Most examined PES in post-lingually implanted adults, with one study focused on pre/perilingual adults. Environmental sound identification (ESI) in quiet using open- or closed-set response format was most commonly used in PES assessment, included in all selected studies. ESI accuracy in CI children (3 studies) and adults (16 studies), was highly variable but generally mediocre (means range: 31–87%). Only two studies evaluated ESI performance prospectively before and after CI, while most studies were cross-sectional. Overall, CI performance was consistently lower than that of normal-hearing peers. No significant differences in identification accuracy were reported between CI candidates and CI users. Environmental sound identification correlated in CI users with measures of speech perception, music and spectro-temporal processing.

**Conclusion:** The findings of this systematic review indicate considerable limitations in the current knowledge of PES in contemporary CI users, especially in pre/perilingual late-implanted adults and children. Although no overall improvement in PES following implantation was found, large individual variability and existing methodological limitations in PES assessment may potentially obscure potential CI benefits for PES. Further research in this ecologically relevant area of assessment is needed to establish a stronger evidence basis, identify CI users with significant deficits, and improve CI users' safety and satisfaction through targeted PES rehabilitation.

## Introduction

Improved perception of environmental sounds (PES) is considered a major benefit of cochlear implantation (Duchesne et al., 2017; McRackan et al., 2017, 2019). Environmental sounds can be defined as non-speech, non-musical sounds in the listener's surroundings that convey information about places, objects, and actions. These sounds can help listeners navigate their surroundings, warn of potential dangers, and provide a sense of aesthetic satisfaction. From avoiding a road collision, answering a doorbell, to enjoying birdsongs or waves crashing on the shore, environmental sounds provide a sense of connection to the environments and enhance awareness of it (Ramsdell, 1978). Outside of the early years of cochlear implant (CI) development and clinical use, however, there has been relatively little research attention to PES in CI users (Tyler and Kelsay, 1990), even as implantation criteria have expanded over time. Relevant findings from early studies with profoundly deaf individuals using first generation CIs with a single or several electrodes may not accurately represent PES performance of more recently implanted individuals. Although qualified CI candidates who are considering implantation are often counseled about increased access to environmental sounds, without a clear evidence basis PES in contemporary CI users remains largely a presumed benefit. To address the knowledge gap in this area of CI outcomes assessment, the present review provides a systematic evaluation of the extant published research on CI users' ability to perceive environmental sounds.

Cochlear implants are the treatment of choice for a growing number of people afflicted with sensorineural hearing loss beyond the therapeutic capabilities of acoustic amplification with hearing aids (NIDCD, 2021). Although CIs were initially approved by the Food and Drug Administration (FDA) for adults with profound hearing loss in both ears (Sladen et al., 2017), today's CI candidates may include adults and children who still retain usable, and sometimes normal hearing in at least one ear (e.g., Benchetrit et al., 2021). Patients with greater overall hearing abilities prior to implantation may expect more from their implants afterwards. In addition to improved speech perception, CI users often expect better perception of music and environmental sounds. Recognizing the importance of research in this area, the National Institutes of Health (NIH) Consensus Development Panel on CIs in Adults & Children highlighted “nonspeech benefits of implantation,” such as PES, as a vital future direction for CI research more than two and a half decades ago (NIH Consensus Conference, 1995). Since that time, however, PES in CI users has remained minimally assessed, and the development of new processing strategies and most common outcome measures of auditory performance in CI users have continued to focus primarily on the speech perception and, to a lesser extent, spectro-temporal processing and music perception (McRackan et al., 2017, 2019; Shekar et al., 2021).

In daily life, PES is central to independence and safety of CI users (Bond et al., 2009; Debruyne et al., 2017; Hamel et al., 2020). Both CI candidates and CI users specifically identify PES as an important contributor to quality of life (QOL) (Tyler and Kelsay, 1990; McRackan et al., 2017, 2019). It has been proposed that PES may explain significant improvements in CI-specific QOL in patients who do not demonstrate proportional speech perception gains with CIs (Capretta and Moberly, 2016; Zaidman-Zait et al., 2017; Moberly et al., 2018; Vasil et al., 2020). Distinct from speech and musical sounds, environmental sounds comprise acoustic byproducts of mechanical interactions of sound-producing objects, such as sounds of machinery or nature, or they can be learned, arbitrary associations between a specific sound and its meaning, such as warning signals and alarms (Shafiro et al., 2020). Outside of the laboratory, environmental sounds tend to occur in the presence of other sounds populating a given auditory scene, and their perception can be affected by both energetic and informational masking (Gygi and Shafiro, 2013; Shafiro et al., 2016). Nevertheless, past research indicates that healthy normal-hearing listeners can readily identify a wide variety of common environmental sounds and can infer detailed information about their sources (Carello et al., 1998; Pastore et al., 2008; Lemaitre and Heller, 2013). Much less is known, however, about PES in contemporary CI users.

This systematic review was designed and conducted to identify and examine published studies of PES in CI users in order to synthesize relevant empirical evidence and appraise existing methods of PES assessment. The review's primary objectives were to (a) ascertain the ability of CI users to perceive environmental sounds and (b) to determine whether PES improves following implantation. To our knowledge, no systematic review in this area has been previously conducted. Given the clinical importance of PES for CI users and limited research in this area of assessment, the inclusion criteria were set broadly to capture as much pertinent research as possible across patient populations, implant models, and assessment methods. To make review findings relevant to contemporary CI users, only studies that provided a quantitative assessment of PES in CI users published in the 21st century were included.

## Methods

The goal of this systematic review was to provide a broad assessment of CI users' abilities to perceive environmental sounds. In addition, the following specific questions were addressed:

(1) Does current evidence indicate an improvement in perception of environmental sounds (PES) following CI?
(2) Does the degree of improvement in PES following CI differ between CI populations (pre-lingual and post-lingual children and adults)?
(3) What are predictors of PES improvement in CI users?
(4) What assessment methods have been used to evaluate PES in CI users?

The protocol for this systematic review was registered with PROSPERO (International Prospective Register of Systematic Reviews, University of York, https://www.crd.york.ac.uk/prospero, Protocol number CRD42021248601).

### Search Strategy

**Figure 1:**
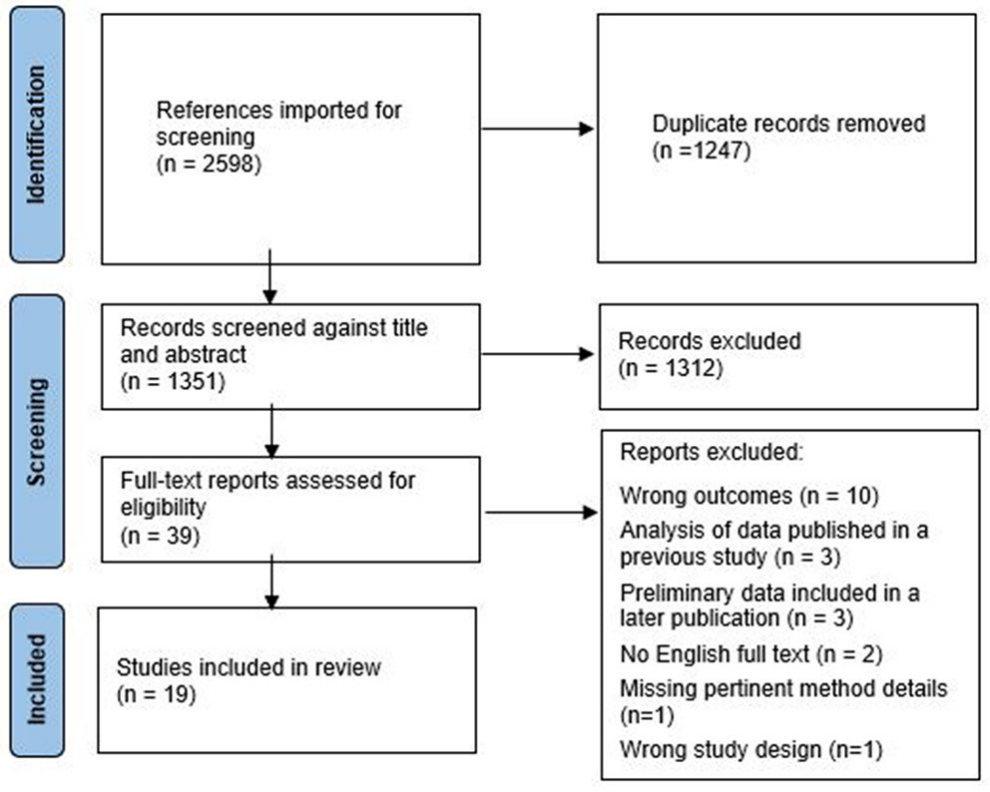
PRISMA flow diagram.

The Preferred Reporting Items for Systematic Reviews and Meta-analyses (PRISMA) Extension for Scoping Reviews (PRISMA-ScR) checklist (Peters et al., 2015; Tricco et al., 2018) was used as the reporting guide for this review (Figure 1). A comprehensive literature search was developed by a medical librarian and reviewed using the Peer Review of Electronic Search Strategies (PRESS) guidelines (McGowan et al., 2016). Searches were conducted in February and March, 2021 in MEDLINE (Ovid), Scopus, Web of Science, Cochrane Library, Cumulative Index to Nursing and Allied Health Literature (CINAHL), and ComDisDome. Searches were limited to articles from 2000 to 2021. The search strategies were created using medical subject headings (MeSH) and keywords combined with database-specific advanced search techniques. MeSH terms and keywords were broadly identified to reference CIs and environmental sounds. The full search strategy is further detailed in Supplementary Material. A total of 2,598 results from the literature searches were saved and imported into Covidence (www.covidence.org), a web application for managing systematic reviews. After 1,247 duplicate entries were removed, the remaining 1,351 were screened by two independent reviewers to determine eligibility for this review. The first phase of screening was a title/abstract review, and potentially relevant articles were moved to the second phase of screening for the full text of the publications. The screening was conducted in Covidence. All conflicts were resolved with group consensus.

### Eligibility Criteria

Study selection was based on Population, Intervention, Control, and Outcomes (PICOS) guidelines (Tacconelli, 2009), summarized in Table 1. Studies were selected if they contained quantitative assessment of PES in CI users of any age, etiology, or duration of hearing loss, all language abilities (pre-lingual/perilingual or post-lingual), with any CI model or hearing modality (unilateral CI, bilateral CI, bimodal). Studies were excluded from the review if they were published prior to the year 2000, were based on single-channel CIs, or assessed PES based solely on anecdotal reports or expert opinions.

**Table 1:** PICOS (Patient, Intervention, Control, Outcome, and Study Design).

P	Adults or children users of cochlear implants
I	Cochlear implantation
C	A control group of normal hearing or hearing impaired peers with or without hearing loss, pre-to-post-implantation comparisons
O	Quantitative assessments of environmental sound perception, associations with speech and auditory processing measures
S	Observational studies: cross-sectional, pre- and post-implantation repeated measures.

### Data Extraction

Information from the full texts of selected studies that met the inclusion criteria was extracted. This information included study design and methods (Table 2), study sample size and subject characteristics (Table 3), type of PES assessment used, and task characteristics (Table 4), as well as correlations to other auditory performance outcome measures (Table 2).

**Table 2:** Characteristics of the studies included in the systematic review.

**Author and year**	**LoE and quality**	**Study design**	**Subjects**		**Assessment**		**Correlations**
			**CI**	**Cntrl**	**PES tests**	**Non-PES auditory tests**	**CI PES and other auditory tests**
Harris et al. (2021)^**^	2b Good	Pre-post	Adult	Self	ID	AzBio; SMRT	Speech ♦, Spectro-temporal ♦
Shafiro et al. (2020)	2b Good	Cross-sectional	Adult	ONH	ID and Serial recall	15 additional tests of speech, music and psychoacoustic spectro-temporal processing	Speech ■♦, Music ♦, Spectro-temporal ●■
McMahon et al. (2018)	2b Good	Cross-sectional	Adult	OHI	ID	AzBio	NR
Strelnikov et al. (2018)	2b Good	Cross-sectional	Adult	–	ID and Ctgrs.	Disyllabic words; Sentences in noise	Speech ♦
Chang et al. (2017)	2b Good	Cross-sectional	Adult	YNH	ID	Vowels, Consonant	Speech ♦
Zhang et al. (2016)	2b^*^ Fair	Cross-sectional	Adult	NH	ID	NR	NR
Shafiro et al. (2016)	2b Good	Cross-sectional	Adult	YNH MON MOI	ID and Serial recall	BKB-SIN	Speech ♦
Shafiro et al. (2015)	2b Good	Cross-sectional	Adult	–	ID	CNC, SPIN-R	Speech ■, ♦
Heo et al. (2013)	2b Good	Cross-sectional	Adult	–	ID Locl.	NR	NR
Shafiro et al. (2011)	2b Good	Cross-sectional	Adult	–	ID	CNC, HINT	Speech ♦
Lee and Kim (2011)	2b^*^ Fair	Cross-sectional	Adult	HA	ID	Monosyllabic words	Speech ♦
Looi and Arnephy (2010)	2b Good	Cross-sectional	Adult	NH	ID	Speech perception (specific test not described)	NR
		Pre-post		Self			
Inverso and Limb (2010)	2b Good	Cross-sectional	Adult	–	ID Ctgrs.	CNC-Words, CNC-Phonemes, HINT-Quiet, HINT-Noise	Speech ●■♦
Kaga and Akamasu (2009)	4 Poor	Cross-sectional	Adult	CD	ID	NR	NR
				AN			
Reed and Delhorne (2005)	4 Poor	Cross-sectional	Adult	–	ID	NU-6	NR
Peasgood et al. (2003)	2b Fair	Cross-sectional	Adult (non-traditional candidates)	–	ID	Speech pattern perception, CUNY sentences	Speech ●♦
Berland et al. (2019)	2b Good	Cross-sectional	Children	NH	ID Ctgrs.	NR	NR
Liu et al. (2013)	2b Good	Cross-sectional	Children	–	ID	PPVT-R vocabulary test	Speech ■
Kim and Lee (2012)	2b^*^ Good	Cross-sectional	Children	NH, HA	ID	Word and sentence recognition (specific tests not described)	Speech ♦

*LoE, levels of evidence; Cntrl., controls; PES, Perception of environmental sounds; NR, not reported; ID, identification; Ctgris., Categorization; Locl., Localization; NH, normal hearing; ONH, older normal hearing; YNH, younger normal hearing; MON, middle/older aged normal hearing; MOI, middle/older aged impaired; HA, hearing aid users; CD, cortical deafness (auditory agnosia); AN, auditory neuropathy; Correlation magnitude symbols: ● = low r <0.3, ■ = medium r = 0.3 ⋜ 0.49, large > 0.5 ♦;*

* *= not published in English;*

** *= correlation symbols reflect synchronous results for a 12-month time point for 11 subjects, to be comparable with other studies in the table*.

**Table 3:** Characteristics of cochlear implant participants across the studies reviewed.

**Author and year**	***N***	**Age (years) mean (range)**	**CI experience (years) mean (range)**	**Language history**	**Modalities tested**
Harris et al. (2021)	20	67 (49–82)	pre-CI, 0.5 and 1	Post-lingual	Bimodal
Shafiro et al. (2020)	40	61 (24–84)	6 (1–29)	Post-lingual	15 bimodal, 17 unilateral, 8 bilateral
McMahon et al. (2018)	39	68 (50–83)	7 (1.5–34)	Post-lingual	12 bilateral, 14 bimodal, 13 unilateral
Strelnikov et al. (2018)	17	60 (46–74)	0.	NR	NR
	15	45 (23–67)	0.8		
	16	56 (41–71)	5		
Chang et al. (2017)	10	45 (19–65)	3.5 (1–4.5)	Post-lingual	Unilateral
Zhang et al. (2016)	9	31 (18–45)	5.1 (0.5–13)	NR	NR
Shafiro et al. (2016)	8	54 (25–68)	3.6 (1.3–9)	Post-lingual	Unilateral
Shafiro et al. (2015)	14	63 (51–87)	5 (1–8)	Post-lingual	Unilateral
Heo et al. (2013)	14	51 (35–66)	1.2 (0.6–2.6)	Post-lingual	Bimodal, Unilateral
Shafiro et al. (2011)	17	58 (40–80)	3.2 (1-7)	Post-lingual	Unilateral
Lee and Kim (2011)	9	35 (24–69)	3	4 post-lingual, 5 pre-lingual	Unilateral
Looi and Arnephy (2010)	10	58 (29–77)	2.3 (0.8–4.8)	Post-lingual	Unilateral
	4	55 (43–66)	pre-CI and 0.25	Post-lingual	Unilateral
Inverso and Limb (2010)	22	59 (39–75)	At least 1 year	Post-lingual	NR
Kaga and Akamasu (2009)	17	50 (14–75)	NR	Post-lingual	NR
Reed and Delhorne (2005)	11^*^	42 (29–67)	6.9 (1-12)	10 post-lingual and 1 pre-lingual	Unilateral
Peasgood et al. (2003)	10	31 (15–52)	3.4 (0.8–6.3)	Pre-lingual	NR
Berland et al. (2019)	24	9 (6–11)	6.3 (0.8–7.6)	Pre-lingual and Early Implanted	Unilateral
Liu et al. (2013)	21	5 (3–6)	1.6	Pre-lingual	NR
	26	8 (6–10)	2.9	Pre-lingual	
Kim and Lee (2012)	22	12 (7–15)	5.7	Pre-lingual	Unilateral

*For the columns “Age” and “CI experience” the average and range are provided in years; N, number of participants; NR, not reported;*

* *only 7 of 11 participants completed all testing*.

**Table 4:** Environmental sound assessment tasks and results.

**Author and year**	**Task**	**Response options**	**Stimuli**	**Group**	**PES result**
Harris et al. (2021)	Identification	25 names	25 sounds (1 token each)	CI (post-test−6 months)	65% (SD = 14.3)
				CI (post-test−12 months)	69.1% (SD = 15.7)
				HI-CIC (pretest)	64% (SD = 14.1)
Shafiro et al. (2020)	Identification	15 names	24 sounds (1 token each)	CI	74% (SD = 16.8)
				ONH	95% (SD = 5)
	Identification and Serial recall	3, 4, or 5 names (based on the number sounds in a given sequence)	24 sounds—sequences of 3, 4, or 5 sounds	CI ONH	59% (SD = 23) 70% (SD = 18)
McMahon et al. (2018)	Identification	25 names	25 sounds (1 token each)	CI	59% (SD = 14.3)
				HI-CIC	55% (SD = 26.4)
Strelnikov et al. (2018)	Identification	Open/3 categories	16 sounds (1 token each, included music)	CI (new users)	33% (SD = 30)
				CI (intermediate users)	35% (SD = 29)
				CI (experienced users)	30% (SD = 17)
	Categorization	Free sorting		CI (new users)	43%
				CI (intermediate users)	55%
				CI (experienced users)	60%
Chang et al. (2017)	Identification	9 names	9 sounds (1 token each)	CI	78.9% (SD = 20.6)
				YNH	98.9% (SD = 3.5)
Zhang et al. (2016)	Identification	16 names	67 sounds	CI	63.18%
				NH	96.16%
Shafiro et al. (2016)	Identification (percent correct sound name regardless of order accuracy)	25 names	20 sounds—sequences of 5	CI	69% (SD = 25)
				YNH	78% (SD = 4.4)
				MON	73% (SD = 11.8)
				MOI	73% (SD = 13.9)
	Serial Recall (percent correct sound names placed in correct order)			CI	45% (SD = 20.1)
				YNH	65% (SD = 8.2)
				MON	44% (SD = 18.2)
				MOI	44% (SD = 20.3)
	Serial recall (percent entire sequences corrects)			CI	14% (SD = 16.9)
				YNH	43% (SD = 11.1)
				MON	14% (SD = 13.4)
				MOI	14% (SD = 18.6)
Shafiro et al. (2015)	Identification	60 names	40 sounds (4 tokens each)	CI	47% (SD = 14.9)
Heo et al. (2013)	Identification	Open	40 sounds (4 tokens each)	CI (bimodal)	36% (SD = 10.3)
				CI (unilateral)	29% (SD = 11.9)
	Localization	8 speakers		CI (bimodal)	75% (SD = 7.4)
				CI (unilateral)	63% (SD = 5.0)
Shafiro et al. (2011)	Identification	60 names	40 sounds (4 tokens each)	CI	45% (SD = 16.2)
Lee and Kim (2011)	Identification	10 names	40 sounds (2 tokens each)	CI	33% (SD = 17.9)
				HI-HA	40% (SD = 19.2)
Looi and Arnephy (2010)	Identification	45 names	45 sounds (2 tokens each)	CI (experienced)	59% (SD = 11.5)
				NH	93% (SD = 4.3)
				CIC	40% (SD = 14.3)
				CI (3 month post-test)	57% (SD = 21.4)
Inverso and Limb (2010)	Identification	Open set	40 sounds (50 total tokens)	CI	48% (SD = 13.5)
	Categorization	5 names			71% (SD = 13.5)
Kaga and Akamasu (2009)	Identification	Open set	24 sounds	CI	42%
				CD	8%
				AN	50%
		4 images		CI	88%
				CD	46%
				AN	92%
Reed and Delhorne (2005)	Identification	10 names	40 sounds (3 tokens each)	CI	79% (SD = 15.5)
Peasgood et al. (2003)	Identification	Open set	20 sounds (1 token each)	CI	41% (SD = 13.7)
Berland et al. (2019)	Identification	Open set	18 sounds (1 token each) —includes musical, vocal, and environmental sounds	CI	35%
Liu et al. (2013)	Identification	4 images	30 sounds (single token)	CI (younger group)	61% (SD = 23.8)
				CI (older group)	73% (SD = 20.5)
Kim and Lee (2012)^*^	Identification	10 images	40 sounds (4 tokens)	CI	31.67%
				NH	96.5%
				HA	30.7%

*CI, cochlear implant; CIC, cochlear implant candidates; NH, normal hearing; ONH, old normal hearing; YNH, young normal hearing; MON, middle/older aged normal hearing; MOI, middle/older aged impaired; HA, hearing aid; CD, cortical deafness (auditory agnosia); AN, auditory neuropathy;*

* *the reported PES score is the average of two similar scores obtained with 5 dB SNR using background noise recorded before the class and after the class*.

### Quality Evaluation and Risk of Bias

The risk of bias for each study was assessed using (a) The Oxford Centre for Evidence-Based Medicine (CEBM) Levels of Evidence guidelines (Oxford Centre for Evidence-Based Medicine, 2009) and (b) the NIH study quality assessment tools (NIH, 2021). These quality evaluation tools provide additional criteria for specific study designs which complement the level of evidence metric and can be used to assign a quality rating of Good, Fair, or Poor.

### Data Analysis

The extracted data were evaluated following synthesis without meta-analysis (SWiM) guidelines (Campbell et al., 2020). SWiM guidelines are consistent with and further expand PRISMA methodology (www.prisma-statement.org) to provide formal guidance for the synthesis of quantitative studies for which meta-analysis cannot be completed. This type of analysis was deemed most appropriate given the large methodological variation in previous PES studies including differences in assessment methods, study design, and populations.

## Results

### Study Characteristics

The 19 selected studies were published between 2003 and 2021. Nine studies were conducted in the United States, four in Korea, two in France, and one at each of the following: the United Kingdom, New Zealand, Taiwan, and China. Sixteen studies were published in English, one in Chinese, and two in Korean. Pertinent details for the three studies not published in English were obtained through translation specifically for this review or provided in personal communications by the study authors. All studies were published in peer-reviewed journals, except (Kaga and Akamasu, 2009), which was published as a book chapter.

Sixteen of the selected studies examined PES in adult CI users and three studies examined PES in children with CIs with congenital or early onset hearing loss (Kim and Lee, 2012; Liu et al., 2013; Berland et al., 2019). Most of the adult CI studies focused on post-lingually implanted adults, with only one of the adult studies (Peasgood et al., 2003) focused exclusively on pre/perilingual CI users. Two additional studies also included pre/perilingual CI users: one had about an equal number of post- and pre/perilingual adults (Lee and Kim, 2011) and the other included only one pre-lingual participant (Reed and Delhorne, 2005).

Study quality, assessed with the NIH-NHLBI study quality assessment tool, was judged “Poor” for two studies, “Fair” for six studies, and “Good” for 11 studies. The 17 studies rated as “Good” or “Fair” were classified as 2b on the Oxford Level of Evidence scale, while the studies of “Poor” quality were classified as 4 on this scale (Oxford Centre for Evidence-Based Medicine, 2009).

### Study Designs

The majority of studies used a cross-sectional design. Two studies utilized a longitudinal pre-to-post implantation paradigm (Looi and Arnephy, 2010; Harris et al., 2021). One of the two longitudinal studies, however, had a small sample of only four participants in its pre-to-post-implantation arm (Looi and Arnephy, 2010). In addition to participants serving as their own control, at least one control group was included in 10 studies, while the remaining studies referenced prior research using the same assessment instruments or otherwise deemed their stimuli to have high or near-ceiling accuracy for healthy individuals with normal hearing. When a control group was used, control listener populations were quite variable across the studies and included, for pediatric studies, children with normal hearing or with a hearing impairment and, for adult studies, adults who were young normal hearing, older normal hearing, older hearing impaired, or had other comorbid conditions affecting auditory processing (auditory neuropathy or cortical deafness).

### CI Participants

A total of 395 CI users (302 adults and 93 children) were evaluated across the selected studies. Sample sizes of CI participants varied considerably (range 8–48) with an average of 20 participants per study. In most studies, participants were experienced CI users, having various hearing loss etiologies and at least a year of CI experience. All were implanted with devices approved for implantation at the study site prior to participation. The average duration of CI experience in cross-sectional studies that reported this value for adults was 4.2 years and for children 4.1 years. Harris et al. (2021) examined CI users at both 6 and 12 months after implantation and three other studies did not report an average duration of CI experience (Kaga and Akamasu, 2009; Inverso and Limb, 2010; Zhang et al., 2016). Performance of CI users with <1 year of CI experience was specifically examined in three studies (Looi and Arnephy, 2010; Strelnikov et al., 2018; Harris et al., 2021). Several studies also included some participants that had 3–12 months of CI experience, although the average duration of CI experience for participants in those studies was considerably longer than 1 year (Peasgood et al., 2003; Looi and Arnephy, 2010; Heo et al., 2013; Liu et al., 2013; Zhang et al., 2016; Berland et al., 2019).

### Assessment Tasks

The most common type of assessment, included in all 19 studies, was environmental sound identification (ESI), in which CI users heard a single environmental sound, and, in two studies, also sequences of several environmental sounds in series. The participants were asked to either provide their own name for the sound they heard in an open-set response format or to select the most appropriate name for the sound from a closed-set of names. When such closed-set response formats were used, there was further variability across studies in the number of response options provided for naming the sound stimuli, ranging from 4 to 60 response options. One study (Reed and Delhorne, 2005) additionally constrained response options by including names of the settings in which sounds could be heard (e.g., “Kitchen,” “Office”). The two studies that used sequences of several environmental sounds to examine CI users' ability to name the sound (Shafiro et al., 2016, 2020), also examined the ability to recall the specific order in which the sounds were presented, thus placing a greater demand on auditory working memory. In addition to sound identification, three studies also examined categorization of environmental sounds either by providing participants with specific category names (Inverso and Limb, 2010) or asking participants to group sounds into categories of their choice in a free sorting task (Strelnikov et al., 2018; Berland et al., 2019). A single study also examined localization of environmental sounds, in addition to identification (Heo et al., 2013).

### Stimuli and Procedures

In all studies, environmental sound stimuli were sourced from publicly available audio recording libraries or online databases, and sometimes included recordings made specifically for the study. The stimuli in most tests tended to be broadly sampled from different categories of meaningful environmental sounds, including sounds of nature, urban environments, machinery, household, alarms and warnings, animal and human non-speech vocalizations or bodily sounds. In some studies (e.g., Inverso and Limb, 2010; Strelnikov et al., 2018), stimuli also included sounds of musical instruments and samples of human speech for judgments of indexical properties.

The number of stimuli in a single test varied between nine (Chang et al., 2017) and 160 (Shafiro et al., 2011), with several studies using multiple sound tokens of the same type of sound (e.g., four different “dog barking” sounds). The maximum number of different types of sounds in one test was 67 (Zhang et al., 2016). In most studies, the test stimuli were presented to participants only once in a single session. In some studies, stimuli were presented more than once for different tasks, for example, first for free sorting of sounds into groups and then for identification (Strelnikov et al., 2018), or when stimuli were modified by different lowpass and highpass filters (Chang et al., 2017). In Zhang et al. (2016), participants could replay the sound up to three times, and in Berland et al. (2019), there was no limit to the number of times the participants could replay the sounds. In two other studies that used sequences of individual environmental sounds on each trial (Shafiro et al., 2016, 2020), participants were first tested on individual sounds and then heard each sound twice but in two different sound sequences.

Most participants were tested at the study sites in a sound booth or a quiet room, with a loudspeaker positioned one-meter away from the participant, presenting stimuli at either a comfortable or a set presentation level (65–70 dB SPL). In one study, a subgroup of participants was tested at home with their preferred audio settings following a calibration with multitalker babble, during which sound levels could be adjusted (Shafiro et al., 2020). Nine studies tested participants with a unilateral CI alone, one tested all participants bimodally, with a CI and a hearing aid, three studies included participants in some mix of three modalities: unilateral, bimodal, bilateral, and six studies did not specify listening modality during testing.

Only one study (Kim and Lee, 2012) examined ESI in the presence of background noise, using a fixed 5 dB signal-to-noise ratio (SNR) and two types of classroom noises recorded either before the class begins or during the break period. In all other studies, environmental sounds were always presented in quiet.

### Accuracy

Identification accuracy scores for isolated environmental sounds differed considerably across the 19 studies. For post-lingual adults, identification accuracy ranged between ~33% correct (Strelnikov et al., 2018) and 87.5% correct (Kaga and Akamasu, 2009). For children with CIs, two studies reported sound identification accuracy of 31.6 and 35.3% (Kim and Lee, 2012; Berland et al., 2019), while a third study reported 67.6% accuracy (Liu et al., 2013). The single study, which focused specifically on pre/perilingual late-implanted adults, reported identification accuracy of 40.5% (Peasgood et al., 2003).

**Figure 2:**
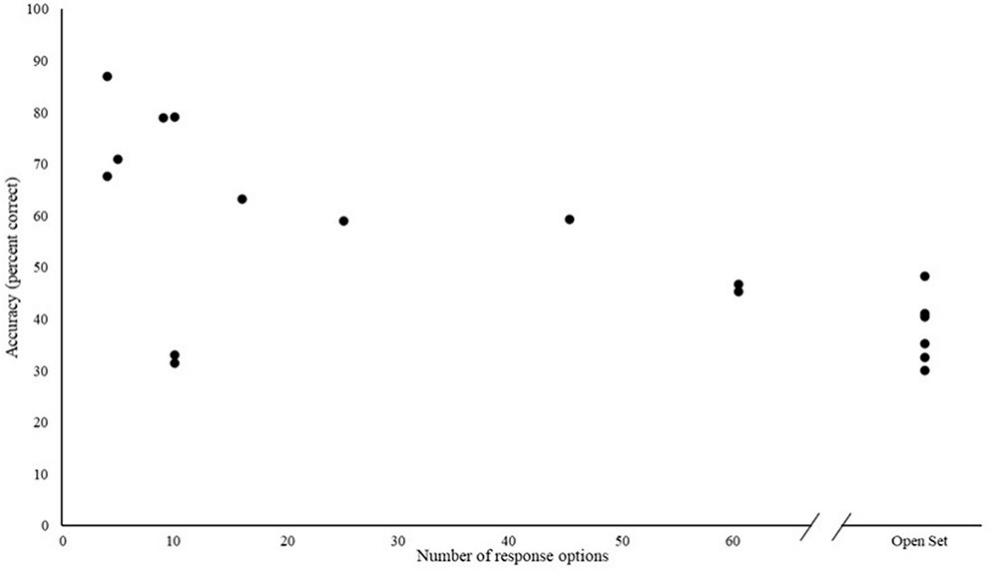
Identification accuracy and the number of response options in a test.

To an extent, such wide variation in ESI accuracy appears to be related to response format. For instance, in one study, when the same environmental sound stimuli that produced 87.5% correct in a 4-alternative forced choice (4AFC) response format were presented to the same CI users in an open set that required them to name each sound, accuracy decreased to 41.7% correct (Kaga and Akamasu, 2009). The general relationship between identification accuracy and response set size is further illustrated in Figure 2. Excluding the five studies with open set responses, there is a negative Spearman Rho correlation of −0.39 (*p* > 0.05). However, if the open set studies are conservatively assigned the value above 60 response options (since all five open set studies reported accuracy which was close to or below that of studies with 60 response options), the rank order correlation magnitude increases to Rho = −0.64 (*p* < 0.01). The two outlier studies in Figure 2 with lower accuracy scores obtained on tests with a relatively small number of response options (i.e., 10; Lee and Kim, 2011; Kim and Lee, 2012) included early deafened participants, some of whom relied primarily on sign language prior to implantation.

Considering the wide variation in ESI scores of CI users, further comparisons with ESI accuracy in control groups provides a useful context for evaluating CI performance. It is notable that when normal hearing controls were included (Looi and Arnephy, 2010; Zhang et al., 2016; Chang et al., 2017; Shafiro et al., 2020), their ESI for isolated sounds was quite high (>90% correct) regardless of the number of response options. The authors of the studies that did not include normal hearing control groups similarly claimed that the stimuli used in the studies were selected to be highly identifiable by normal hearing listeners, as confirmed through pilot testing or in prior research. Furthermore, when control groups comprised individuals with hearing loss (Kaga and Akamasu, 2009; Looi and Arnephy, 2010; Lee and Kim, 2011; Kim and Lee, 2012; Shafiro et al., 2016; McMahon et al., 2018; Harris et al., 2021), study findings did not reveal significant differences in environmental sound identification accuracy between hearing impaired adults and CI users. The only exception was Kaga and Akamasu (2009), who found a better ESI performance in CI users compared to individuals with cortical deafness (auditory agnosia), while CI users performed similarly to individuals with auditory neuropathy included in the same study. Although not all of the control participants with hearing loss could be considered CI candidates and some may have had milder hearing loss, the lack of significant differences in any of these seven studies is concerning since it indicates no overall ESI improvement following implantation. Notably, in three of the above studies where controls were known to meet CI candidacy criteria (Looi and Arnephy, 2010; McMahon et al., 2018; Harris et al., 2021), likewise, no significant differences in ESI between CI candidates and CI users were observed.

Higher accuracy among CI users was obtained when participants were asked to categorize environmental sounds rather than to identify each sound individually. In one study of experienced adult CI users (Inverso and Limb, 2010), environmental sounds identified individually with 48.3% accuracy in open set naming were categorized with 71.1% accuracy when participants were offered to choose from five category names for each sound. As with individual sounds, however, the increase in accuracy scores could be also related to the reduction in the number of response options. Nevertheless, in another study where three predefined categories were applied to sound groupings created by CI users themselves in a free sorting task using 16 individual sounds (Strelnikov et al., 2018), categorization accuracy varied between 43%, in the first 3 months of CI use, to 60% for patients with more than 12 months of CI experience. In contrast, identification accuracy for the same sounds individually ranged from 30 to 35% across the three CI experience groups.

Accuracy scores were also affected when more than one environmental sound was presented on a single trial. For example, in Shafiro et al. (2016), when asked to identify five sounds presented together in a specific order by selecting sound names from a set of 25 response options, whole sequence identification accuracy was 14%, for placing correct sound names in the correct presentation order. In a follow up study, when Shafiro et al. (2020) modified the number of sounds in each sequence to match the number of sounds in the stimulus sequence, overall accuracy rose to 59%.

Another factor that seems to have influenced CI users' environmental sound identification scores was listening modality. However, only a couple of studies reported scores based on the CI listening modality. McMahon et al. (2018) found bimodal CI listeners performed similarly to bilateral CI listeners (64.6 and 63.7%, respectively), while both groups significantly outperformed unilateral listeners (51.4%) on environmental sound identification. These findings were more recently confirmed by Nyirjesy et al. (2020), who expanded the participant pool from the McMahon et al. (2018) study from 39 experienced CI users to 50. Similarly, McMahon et al. (2018) reported that bimodal and bilateral CI users achieved scores of 65.8 and 63.7%, respectively, outperforming unilateral CI users who scored 55.4% correct on the same 25-alternative forced choice test. Heo et al. (2013) reported smaller sound identification differences of 35.5 and 29.5% correct for bimodal and unilateral adult CI users, respectively. It is possible that the smaller differences might have resulted from score range compression due to the overall lower identification scores. In the same study, somewhat larger modality differences were also observed for CI participants for localizing environmental sounds in space with accuracy scores of 74.9% for bimodal and 63.2% for bilateral CI users. Thus, overall, it appears that bimodal and bilateral CI users have some advantage in ESI compared to unilateral CI users.

### Correlations With Speech Perception and Other Auditory Abilities

Correlation analyses of ESI scores with speech and other measures of auditory function were performed in all but three studies (Reed and Delhorne, 2005; Kaga and Akamasu, 2009; Zhang et al., 2016). Note that Reed and Delhorne (2005) did not perform correlation analysis but rather observed that “[p]erformance on the environmental-sound identification test was roughly related to [Northwestern University-6] NU-6 word recognition ability.” In this study, those who scored higher than 34% correct on monosyllabic NU-6 words scored higher on environmental sound identification. When conducted, correlation analyses were based on test scores collected synchronously around the same time period, except in one study (Harris et al., 2021), which also examined the associations between pre-CI and post-CI performance for environmental sounds, speech, and spectral-temporal processing test scores. Because the studies tended to have relatively small sample sizes with a large intra-subject variance typical of CI listeners and used several scoring metrics, the foregoing discussion will focus on correlation magnitudes (Cohen, 1988) that may help to reveal converging patterns across studies.

Correlations between ESI and various measures of speech perception abilities were performed in 13 studies (Table 2). In the majority of these studies, speech materials were presented in quiet, and in five studies were also presented in noise. Across the 13 studies in which associations between environmental sound identification and speech perception in quiet and/or in noise were examined, all 13 studies reported correlations of moderate-to-large magnitude (i.e., *r* > 0.3) with two of the 13 studies also reporting small correlation magnitudes for additional measures of speech perception (i.e., *r* <0.3) (Peasgood et al., 2003; Inverso and Limb, 2010). In one study, ESI was also examined in relation to indexical properties of speech, gender, and emotion identification, reporting moderate to large effect sizes for each (Shafiro et al., 2020). In the same study, Shafiro et al. also reported correlations between PES and music perception (i.e., musical instrument and genre identification) with large effect sizes for both. The associations between ESI and spectro-temporal processing abilities were examined in three studies (Shafiro et al., 2011, 2020; Harris et al., 2021). Across the three studies, correlation magnitudes were distributed between small and large depending on the type of test and also, for Harris et al. (2021), across time-points of analysis relative to the time of implantation.

## Discussion

This systematic review examined published studies of PES in CI users, a perceptual ability which is generally considered to be highly valuable in daily living and an important benefit of implantation. Only studies published since the year 2000 were included to reflect performance of contemporary CI users with multichannel devices. The search strategy and inclusion criteria for the present review were broadly set to allow for the maximal inclusion of any published quantitative assessment of environmental sound perception regardless of participant age, hearing loss etiology, implant type or language and communication background.

The majority of the 19 studies that met the inclusion criteria focused on post-lingually implanted adults. One study focused on pre/perilingual adults and three focused on children with CIs. The most common assessment method used in all studies was ESI, although several studies also included categorization, localization and serial recall. Study results, based primarily on ESI, consistently indicate (1) marked deficits in CI users in comparison to normal-hearing peers, regardless of participant age and language learning background, (2) lack of evidence indicating an overall improvement in ESI following implantation, (3) similar performance across different CI populations, (4) a tendency for bimodal and bilateral CI users to outperform unilateral CI users, and (5) mostly moderate-to-high correlations of ESI with other auditory abilities, including speech and music perception and spectro-temporal processing. This review also highlighted significant limitations in the breadth and depth of research in this area of CI outcomes assessment. Given the recognized ecological importance of PES, the present findings underscore the need for further investigation.

The limited knowledge regarding PES in contemporary CI users is concerning because both the eligibility criteria for implantation and implant technology have changed considerably over the decades (Varadarajan et al., 2021). These changes make extrapolation from earlier studies problematic. Unlike CI patients implanted in the 1980s and 1990s, the majority of whom were profoundly deaf in both ears, today's CI candidates often include adults and children who still retain usable, and in case of single-sided deafness, normal hearing in one ear (Benchetrit et al., 2021). Patients with greater overall hearing abilities prior to implantation may expect more from their hearing after implantation, and post-implantation PES scores that indicated an improvement in the past may no longer be sufficiently high. Nevertheless, evidence from studies of speech perception in contemporary CI users consistently indicate an overall improvement in speech recognition performance following implantation, particularly in quiet and, to a lesser extent in noise (Zwolan et al., 2014; Kelsall et al., 2020; Harris et al., 2021). In contrast, the small number of studies that have investigated PES in contemporary CI users do not indicate a comparable improvement in PES following implantation. Although the results are limited by the reliance on ESI as the primary PES assessment method, they reveal a generally mediocre performance and a large variability in CI users' performance, even for environmental sounds presented in quiet.

Overall, the present findings contrast with commonly held clinical views and anecdotal reports that environmental sound perception improves following implantation. Reasons for this apparent contradiction may reflect the large variability in PES performance levels of individual CI users, limitation in assessment methods and the general lack of clinical and research attention to PES as a post-CI assessment area. It is possible that following implantation, CI patients who can successfully recognize new or previously inaudible sounds are more likely to share their positive experience than those who have no or marginal changes in PES. That is, the lack of awareness in environmental sound recognition may be less readily apparent to the CI user compared to difficulties in recognition of speech, which tend to be overt and obvious – oral language users are usually well-aware when their speech perception is disrupted and they are not able to understand the words of another talker. However, CI candidates who had limited access to environmental sounds prior to implantation, often for extended periods of time, may not realize that they still cannot recognize many common environmental sounds unless they are specifically asked about it or formally tested.

The apparent discrepancy between research findings and anecdotal clinical experiences with respect to PES in CIs may also result from limitations in the assessment methods used to examine PES. The most common type of assessment administered across the studies was identification of isolated environmental sounds presented in quiet. There was also a large variation across studies in the rigor of stimulus development and selection, the number of the stimuli and the number of response options. The wide range of identification accuracy scores from different tests can give a skewed sense, especially since the number of response options used in closed set identification may influence the result (Figure 2) and because certain environmental sounds, such as those with strong temporal patterning, may be inherently more identifiable to CI users' than others (Reed and Delhorne, 2005; Shafiro, 2008a,b). Thus, without rigorous sampling, some stimulus sets used in ESI tests may contain inherently more or less identifiable sounds, biasing the overall outcome.

Furthermore, in everyday ecological encounters environmental sounds are rarely heard in isolation and tend to be accompanied by some contextual cues. Listeners are usually aware of the environment they are in and can leverage situational context and information from other sensory modalities to optimize PES. However, only one study (Kim and Lee, 2012) has examined environmental sound identification in the presence of background noise, while two studies have assessed the effect of context in sequences of environmental sounds distinguished by their semantic coherence with each other (Shafiro et al., 2016, 2020). Although environmental sound identification does not appear to improve following implantation, CIs may still positively contribute to environmental sound awareness, for example by informing the listener that something is happening in the environment, which may in turn lead to more accurate source identification when supplemented by visual or other contextual cues.

In natural settings, outside of the laboratory, it is also quite common for environmental sounds to be in motion, rather than stationary (e.g., a car driving by). However, perception of motion in environmental sounds was not investigated in any of the 19 studies included in the present review. Only one of these studies by Heo et al. (2013) investigated environmental sound localization. However, in Heo et al. environmental sounds were presented from one of eight stationary locations evenly distributed around the listener. A more recent study (Bahadori et al., 2021), published after the current literature search was completed, investigated judgments of distance of moving sound objects for two environmental sounds distinguished by their emotional content – either negative (car wreck) or positive (applause). The judgments of distance were modulated by the emotional content of sounds for 30 normal hearing adults, but not for 10 unilateral CI participants. On the other hand, the authors found a generally comparable ability to localize sounds in space for the CI users and normal hearing controls. Therefore, it is conceivable that following implantation, listeners with CIs may develop improved awareness of objects and events, be more likely to broadly categorize sounds and more accurately perceive the nature of interacting objects and materials, even as their identification accuracy for specific environmental sounds does not improve. With the exception of several studies that examined environmental sound categorization (Inverso and Limb, 2010; Strelnikov et al., 2018; Berland et al., 2019), other potential CI benefits for PES might not be reflected in the existing body of research.

The present review has further revealed that PES assessment is particularly lacking for two CI populations: pre/perilingual late-implanted adult CI users and children. Only one study (Peasgood et al., 2003), published nearly two decades ago, focused exclusively on PES in pre/perilingual adults. The lack of attention to this CI population is surprising given that environmental sound awareness is often one of the main reasons pre/perilingually deafened adults elect to undergo implantation, despite limited expectations about speech perception. Peasgood's et al. (2003) findings are reassuring since pre/perilingual adults in that study demonstrated environmental sound identification scores comparable to those obtained in post-lingual CI users. However, it is worth noting that six of the 10 participants were exclusively aural language users and eight were continuous hearing aid users from their first hearing loss diagnosis through CI surgery. Thus, it remains unclear how much the study findings are applicable to pre/perilingual adults with lesser oral language experience or even more limited access to sound. More research specifically focusing on more recently implanted pre/perilingual adults is needed to estimate their performance and inform pre-CI counseling in this population.

Similarly, only three studies that met this systematic review's inclusion criteria examined PES in children (Kim and Lee, 2012; Liu et al., 2013; Berland et al., 2019). All three studies demonstrated substantial deficits in ESI and categorization for the pediatric population. Two studies demonstrated a low performance of ~30–35%, on average, while the higher score of 61–73% in CI children in the remaining study was obtained in a 4AFC format, while the CI results were still lower than normal hearing peers of the same chronological age. Partly, the low number of PES reports for children may reflect the greater difficulty of administering quantitative tests in this population, combined with the paucity of available tests. It is important to note that several studies that reported on PES in children were not included in the present review because they provided only anecdotal reports and clinician impressions or used rating scales that were not specific to environmental sounds. Although it is possible that, similar to adults, the limited available quantitative assessments do not capture all PES benefits of CI in children, PES remains an area of concern in this population and may benefit from more targeted intervention (Liu et al., 2013).

Surprisingly, only two studies have prospectively examined PES (using ESI) comparing pre- and post-CI performance in post-lingually implanted adults. However, one of these studies (Looi and Arnephy, 2010) tested only four participants in the pre-post-study arm, while the other (Harris et al., 2021) had a larger but still relatively small sample (20 participants at 6-months post-CI and 11 participants at 12-months). Furthermore, all participants in Harris et al. (2021) were bimodal CI users, which may have also affected their performance (Nyirjesy et al., 2020). Neither study found a significant overall improvement in ESI scores compared to pre-CI performance. Both studies, however, reported considerable individual variation in performance. Thus, one goal for future research in PES among CI users is to determine factors that may distinguish patients for whom PES improves from those for whom it does not. Another important goal for future research is to broaden the range of assessment methods used to evaluate potential PES benefits. An evaluation of other ecologically relevant aspects of PES in addition to ESI, and the role of attention, memory and other cognitive abilities, can lead a fuller understanding of potential PES benefits for CI users and indicate areas of strength and weakness. These may include awareness and recognition of events and objects in naturalistic auditory scenes, ability to recognize action and material properties of sound sources, integration of contextual cues provided by vision and/or other sensory modalities, judgments of location, distance and motion of common environmental sounds, perception of emotional aspects and the ability to recognize specific safety-relevant sounds.

The present systematic review considered research studies published between 2000 and 2021 that quantitatively examined environmental sound perception in CI users. Despite the generally recognized importance of environmental sound perception for individual safety, quality of life, and well-being (McRackan et al., 2017, 2019; Vasil et al., 2020), research in this area of assessment of CI users' performance appears to be significantly lacking. The 19 reviewed studies revealed generally mediocre levels of environmental sound identification and an apparent lack of improvement in group performance following implantation relative to pre-CI baseline. A wide variation in PES ability among CI users was also observed. Importantly, sounds that pose perceptual difficulty for CI users are distributed quite broadly in terms of their acoustic and semantic properties (Inverso and Limb, 2010; Shafiro et al., 2011, 2020; McMahon et al., 2018), and identification of sounds relevant to individual safety is not significantly different from that of non-safety relevant sounds (Hamel et al., 2020; Luzum et al., 2021). On the other hand, PES assessment methods used in the reviewed studies may not have captured some important aspects of environmental sound perception relevant to daily living of CI users. The lack of widely used validated tests that tap into different aspects of environmental sound perception may thus be a major contributing factor to the limited knowledge in this area of CI performance. Thus, strong conclusions about CI users' PES abilities seem premature. A comprehensive assessment of environmental sound perception in the post-implantation follow up can help to identify CI users with PES deficits and serve as an important step toward developing effective rehabilitation.

## Data Availability Statement

The original contributions presented in the study are included in the article/Supplementary Material, further inquiries can be directed to the corresponding author/s.

## Author Contributions

VS, MH, AM, and NL designed the study, discussed the results, and wrote the paper. NL, VS, and MH performed the paper search, paper selection, and data extraction. All authors have read and approved the publication of the final manuscript.

## Funding

This study was supported by: (1) the National Institutes of Health, National Institute on Deafness and Other Communication Disorders Career Development Award 5K23DC015539-02 and the American Otological Society Clinician-Scientist Award to AM; (2) the Triological Society Research Career Development Award and the National Institutes of Health (NIH)/National Deafness and Other Communication Disorders (NIDCD) R21 (1R21DC018871-01A1) to MH.

## Conflict of Interest

The authors declare that the research was conducted in the absence of any commercial or financial relationships that could be construed as a potential conflict of interest.

## Publisher's Note

All claims expressed in this article are solely those of the authors and do not necessarily represent those of their affiliated organizations, or those of the publisher, the editors and the reviewers. Any product that may be evaluated in this article, or claim that may be made by its manufacturer, is not guaranteed or endorsed by the publisher.
