# Supplementary material for: Perception of Environmental Sounds in Cochlear Implant Users: A Systematic Review
Source: Front Neurosci. 2022 Jan 10;15:788899. doi: 10.3389/fnins.2021.788899 (PMC8785216; doi:10.3389/fnins.2021.788899)
Supplement: Supplementary file 1 [file Data_Sheet_1.PDF]

Search strategies, databases and terms used in “Perception of environmental sounds in cochlear implant users: A systematic review” by Valeriy Shafiro, Nathan Luzum, Aaron C Moberly, Michael S. Harris

Database: Ovid MEDLINE(R) and Epub Ahead of Print, In-Process, In-Data-Review & Other Non-Indexed Citations and Daily

<1946 to March 15, 2021>

Search Strategy:

1 cochlear implants/ or ((cochlear adj3 implant*) or (auditory adj3 prosthes*) or (cochlear adj3 prosthes*)).mp.

(17396)

2 exp environment/ and exp sound/ (22668)

3 (environment* adj3 sound*).mp. (2076)

4 ((non-linguistic* or non-speech* or non-voice* or non-vocal* or ecologic* or meaningful or safety relevan* or

warning* or alarm* or nature) adj3 sound*).mp. (1377)

5 2 or 3 or 4 (25683)

6 1 and 5 (985)

7 limit 6 to yr="2000 -Current" (876)

Database: Scopus

(TITLE-ABS-KEY ((cochlear W/3 implant*) OR (auditory W/3 prosthes*) OR (cochlear W/3 prosthes*))) AND ((TITLE-ABS-KEY ((environment* W/3 sound*))) OR (TITLE-ABS-KEY (((non-linguistic* OR non-speech* OR non-voice* OR non-vocal* OR ecologic* OR meaningful OR "safety relevan*" OR warning* OR alarm* OR nature) W/3 sound*))))

192 hits

Database: Web of Science Databases= WOS, BCI, CCC, DRCI, DIIDW, KJD, MEDLINE, RSCI, SCIELO, ZOOREC

(Full titles of Web of Science databases: Web of Science Core Collection, Biosis Citation Index, Current Contents Connect, Data Citation Index, Derwent Innovations Index, KCI Korean Journal

Database, MEDLINE, Russian Science Citation Index, SciELO Citation Index and Zoological Record)

6

277

#4 AND #1

Refined by: PUBLICATION YEARS: (2021 OR 2010 OR 2020 OR 2009 OR 2019 OR 2008 OR 2018 OR 2007 OR 2017 OR 2006 OR 2016 OR 2005 OR 2015 OR 2004 OR 2014 OR 2003 OR 2013 OR 2002 OR 2012 OR 2001 OR 2011 OR 2000)

5

345

#4 AND #1

4

53,439

#3 OR #2

3

36,576

TOPIC: (((non-linguistic* OR non-speech* OR non-voice* OR non-vocal* OR ecologic* OR meaningful OR "safety relevan*" OR warning* OR alarm* OR nature) NEAR/3 sound*))

2

17,385

TOPIC: ((environment* NEAR/3 sound*))

1

28,769

TOPIC: ((cochlear NEAR/3 implant*) OR (auditory NEAR/3 prosthes*) OR (cochlear NEAR/3 prosthes*))

Database: Cochrane Library

Search Name: cochlear implants Sound

Date Run: 17/03/2021 05:00:53

ID	Search	Hits
#1	MeSH descriptor: [Cochlear Implants] explode all trees	137

#2 ((cochlear near/3 implant*) or (auditory near/3 prosthes*) or (cochlear near/3 prosthes*)):ti,ab,kw 506

#3 #1 OR #2 506

#4 MeSH descriptor: [Environment] explode all trees 13048

#5 MeSH descriptor: [Sound] explode all trees 678

#6 #4 AND #5 351

#7 (environment* near/3 sound*):ti,ab,kw 79

#8 ((non-linguistic* or non-speech* or non-voice* or non-vocal* or ecologic* or meaningful or safety NEXT relevan* or warning* or alarm* or nature) near/3 sound*):ti,ab,kw 145

#9 #6 OR #7 OR #8 557

#10 #3 AND #9 with Cochrane Library publication date Between Jan 2000 and Mar 2021 17

Database: CINAHL (Cumulative Index to Nursing and Allied Health Literature)

S10 S3 AND S9 Limiters - Published Date: 20000101-20210331

583

S9 S6 OR S7 OR S8

8,818

S8 ((non-linguistic* or non-speech* or non-voice* or non-vocal* or ecologic* or meaningful or "safety relevan*" or warning* or alarm* or nature) N3 sound*)

476

S7 (environment* N3 sound*)

518

S6 S4 AND S5

8,000

S5 (MH "Sound+")

9,943

S4 (MH "Environment+")

140,765

S3 S1 OR S2 E

S 10,717

S2 ((cochlear N3 implant*) or (auditory N3 prosthes*) or (cochlear N3 prosthes*))

10,717

S1 (MH "Cochlear Implant+") OR (MH "Cochlear Implant Programming")

9,323

Database: ComDisDome (searched on March 26, 2021)

(TITLE-ABS-KEY ((cochlear W/3 implant*) OR (auditory W/3 prosthes*) OR (cochlear W/3 prosthes*))) AND ((TITLE-ABS-KEY ((environment* W/3 sound*))) OR (TITLE-ABS-KEY (((non-linguistic* OR non-speech* OR non-voice* OR non-vocal* OR ecologic* OR meaningful OR "safety relevan*" OR warning* OR alarm* OR nature) W/3 sound*))))

146 results
